# Heterogeneity of estrogen and β-adrenergic receptors in female human coronary artery endothelial cells

**DOI:** 10.1016/j.ijcha.2026.101938

**Published:** 2026-05-15

**Authors:** Basant Elsaid, Irakli Kopaliani, Ansam Seif, Stephan Speier, Andreas Deussen

**Affiliations:** a Institute of Physiology, Faculty of Medicine, Technische Universität Dresden, Dresden, Germany; b Department of Physiology, Faculty of Medicine, Ain Shams University, Cairo, Egypt

**Keywords:** ERα, ERβ, GPER, β_1_-AR, β_2_-AR, β_3_-AR

## Abstract

**Introduction:**

Estrogen protects vascular function, likely via adrenergic modulation. Previous studies of estrogen and beta-adrenoceptor (β-AR) interactions relied on non-coronary or animal models, limiting relevance to human coronary endothelium. This study aimed to characterize estrogen receptor (ER) and β-AR expression in human coronary artery endothelial cells (HCAECs), evaluate inter-donor variability, and determine whether ER stimulation alters β-AR gene expression.

**Methods:**

HCAECs from female donors were analyzed for expression of ERs (ERα, ERβ, GPER), and β-ARs (β_1_-, β_2_-, β_3_-AR) at mRNA and protein levels using RT-qPCR, western blotting, and immunofluorescence, with assessment of inter-donor variability. To investigate estrogen–β-AR interactions, cells were treated with 1 and 10 nM 17β-estradiol or 10 nM selective ER agonists; PPT (Propylpyrazoletriol, ERα), DPN (Diarylpropionitrile, ERβ), G-1 (G protein-coupled estrogen receptor, GPER) for 4, 12, and 24 h, and β-AR mRNA expression was measured.

**Results:**

Immunofluorescence revealed presence of all three ERs and β-ARs in the HCAECs. ERα had the lowest mRNA expression, while GPER was the most abundant, with donor-dependent variability. ERβ and GPER proteins were slightly higher than ERα. β_3_-AR exhibited the lowest mRNA, β_2_-AR was the highest, and donor-specific differences were observed. Stimulation with 17β-estradiol or selective ER agonists did not alter β-AR mRNA expression.

**Conclusion:**

These findings demonstrate donor variability in ER and β-AR expression in HCAECs, limiting generalization. Under the tested conditions estrogen and ER agonists did not alter β-AR mRNA levels, suggesting that short-term ER stimulation is unlikely to regulate β-AR expression at the transcriptional level within this experimental setting.

## Highlights

• Female HCAECs express different subtypes of estrogen receptors and β-adrenergic receptors.
• ERα shows the lowest expression among ERs, while GPER is the most abundant.
• Among β-AR, β3-AR shows the lowest expression, while β2-AR is the most highly expressed.
• Significant inter-donor variability exists in both ER and β-AR mRNA and protein expression.
• Short-term 17β-estradiol or selective ER agonists do not alter β-AR mRNA in female HCAECs.

## 1 Introduction

Cardiovascular diseases (CVDs) pose a significant global health challenge, with women generally experiencing lower mortality than men [1]. Postmenopausal women face a twofold increase in cardiovascular risk, largely due to hypertension (HTN), contributing to higher stroke and ischemic heart disease (IHD) rates [2]. IHD in women is often associated with microvascular dysfunction and coronary tone abnormalities [3], [4]. Despite the widespread use of hormone replacement therapy (HRT), it is linked to elevated risks of stroke, myocardial infarction (MI), and thromboembolic events [5], with estrogen during pregnancy or oral contraceptive use also increasing the risks of HTN, stroke, and spontaneous coronary artery dissection (SCAD) [3], [4], [6].

Female sex hormones can be categorized into estrogens and progestins. Estrogens include estrone (E1), estradiol (E2), estriol (E3), and estetrol (E4), with E2 being predominant during reproductive years, E3 and E4 during pregnancy, and E1 post-menopause [7], [8]. ERs, including nuclear ERα and ERβ, and membrane-bound forms like mERα, mERβ, and GPER (GPR30), are crucial for estrogen signaling [9], [10]. ERα and ERβ, encoded by separate genes, show tissue-specific expression patterns [10]. GPER, with its seven α-helical transmembrane domains, interacts with G-proteins to activate pathways such as cAMP and calcium modulation [5]. Recent studies suggest intercommunication between nuclear and membrane-bound receptors, expanding the complexity of estrogen’s effects [11].

Estrogen exerts genomic effects via DNA interaction through nuclear ERs, and non-genomic effects through membrane-bound receptors (GPER, mERα, mERβ) initiating rapid signaling cascades [6]. These receptors are critical for vascular health, with ERα and ERβ expressed in endothelial and vascular smooth muscle cells across various blood vessels, showing higher receptor density in females than males. GPER is also present in human and rodent vascular tissues [3], [5], [8], [12], [13], [14].

β-ARs are integral components of cardiovascular regulation, with the β_1_-AR, β_2_-AR, and β_3_-AR subtypes contributing to various vascular responses. These receptors are encoded by distinct genes, with β_1_- and β_2_-ARs being intronless, while the β_3_-AR gene contains an intron, producing splice variants without functional differences [15]. While the debate persists over the potential existence of a fourth subtype, β_4_-AR, its role remains under investigation [2]. β_2_-ARs are predominantly expressed in human peripheral arteries, rat large pulmonary arteries and retinal arterioles [2], [16], [17], whereas β_1_-ARs are also present in rat retinal arterioles [17]. β_3_-ARs are found in human coronary microvascular endothelium, rat renal afferent arterioles, large pulmonary arteries, and chicken basilar arteries [16], [18], [19], [20], [21].

The regulation of blood pressure by the sympathetic nervous system (SNS) exhibits marked sex differences. Female rats’ blood vessels tend to exhibit less constriction and greater relaxation in response to catecholamines compared to males, with the endothelial function playing a critical role in mediating these differences [22]. Ovariectomy (OVX) abolishes these sex differences, while estrogen replacement therapy restores vascular responsiveness, particularly through its effects on β_1_- and β_3_-AR [23]. Furthermore, sex-related variations in vascular tone regulation become more apparent in the transition from premenopausal to postmenopausal states. In premenopausal women, β_2_-AR stimulation leads to greater increases in blood flow compared to postmenopausal women, indicating a decline in vasodilatory capacity due to estrogen loss [24]. For postmenopausal women, the benefits of HRT in cardiovascular health are partially attributed to its ability to restore balance in SNS function and normalize β-AR signaling [2]. Moreover, estrogen treatment has been shown to increase β_2_-AR mRNA levels in the mesenteric arteries of rats [25]. In heart, estrogen supplementation in rats has been linked to enhanced β-AR expression, particularly in conditions like ischemia–reperfusion injury and arteriovenous fistula [18], [26].

In endothelial cells, estrogen influences β-AR activity through direct modulation of receptor signaling pathways or by inducing adaptive changes that enhance vascular responses over time. This complexity is further compounded by the localization of ERs and β-ARs in specialized cell membrane domains known as caveolae. These microdomains facilitate specific receptor interactions, affecting function and signaling. For instance, β_3_-ARs and ERα interact with caveolin-1. The signaling mechanisms involving β-ARs and ERs are closely integrated within the GPCR system, with interactions between Gαs and Gαi proteins, facilitating downstream effects on cellular function [11]. Additionally, GPR30 shares structural similarities with β-ARs, enhancing their functional overlap and interconnectivity [27].

Most previous studies investigating estrogen and β-AR interactions have been conducted in non-coronary endothelial cells, often in animal models, which may not accurately reflect the signaling characteristics of the human coronary endothelium. The human coronary endothelium critically involved in cardiovascular function and disease, remains largely unexplored due to limited tissue availability and ethical constraints. Samples are typically obtained from donors who died of cardiovascular disease, which does not reflect normal physiological conditions. To address this gap, we aimed to comprehensively characterize ER and β-AR expression in HCAECs by using cells from younger donors whose deaths were accidental and who had no history of cardiovascular disease or other relevant comorbidities. We also sought to evaluate inter-donor variability in ER and β-AR expressions to assess the biological variability among different donors to provide a basis for the design and interpretation of future studies. Finally, we explored whether ER-stimulation results in a change of β-AR mRNA expression in these cells.

## 2 Materials and methods

### 2.1 Female HCAECs culture

HCAECs were obtained from healthy female donors aged 14 years (Cell Biologics, PB-H-6093), 24 years (Lonza, CC-2585, 21TL316169), and 37 years (Lonza, CC-2585, 20TL365545), all of whom had no recorded history of cardiovascular disease, diabetes, smoking, or vascular pathology; the cause of death in all cases was accidental. Cells were cultured in endothelial cell growth medium MV2 (Cell Biologics, M2268) supplemented with 5% fetal calf serum (FCS), epidermal growth factor (EGF, 5 ng/mL), fibroblast growth factor (FGF, 10 ng/mL), insulin-like growth factor (IGF, 20 ng/mL), vascular endothelial growth factor (VEGF, 0.5 ng/mL), ascorbic acid (1 μg/mL), hydrocortisone (0.2 μg/mL), and 1% penicillin–streptomycin. Cultures were maintained in gelatin-coated T25 flasks at 37°C in a humidified atmosphere containing 5% CO_2_, following the Cell Biologics-manufacturer’s protocol.

Medium was changed every 48 h until ∼70% confluency, then daily. Subculturing was performed at 80–90% confluency using 0.05% Trypsin-EDTA, followed by centrifugation (120 *g*, 5 min). For cryopreservation, cells were resuspended in freezing medium (50% FBS, 10% DMSO, 40% culture medium), gradually frozen at –80°C (Mr. Frosty™, Nalgene) and stored in liquid nitrogen.

### 2.2 Immunofluorescence staining of ERs and β-ARs in female HCAECs

HCAECs from female donors aged 14, 24, and 37 years used at passages ten (P10) and nine (P9), respectively, were seeded onto 8-chamber polystyrene vessel tissue culture-treated glass slides (1.0 cm^2^ per well; Corning® Falcon® Culture Slide, 354118). For each donor, two of these glass slides were seeded with cells. Upon reaching 80–90% confluence, the IF staining procedure was performed according to the method described by Hubert et al. [28]. Cells were fixed for 10 min with 4% paraformaldehyde (PFA), followed by three 10-minute washes with PBS. One slide from each donor underwent permeabilization for 10 min using freshly prepared permeabilization buffer (PBS, 5% FBS, 0.01% Tween-20, 0.75% glycine, 0.1% Triton X-100, filtered through a 0.22 μm membrane), while the second slide was incubated with PBS alone. To reduce nonspecific antibody binding, cells were blocked using protein block serum-free (DAKO, X0909). After blocking, the cells were incubated with primary antibodies diluted in antibody diluent with background-reducing components (DAKO, S3022) overnight at 4°C. The primary antibodies and their respective dilutions were: ERα mouse monoclonal antibody (1:50, Thermo Fisher Scientific, MA1-111), ERβ rabbit polyclonal antibody (1:100, Abcam, ab3575), GPR30 rabbit polyclonal antibody (1:100, Thermo Fisher Scientific, PA1-049), β1-AR rabbit polyclonal antibody (1:100, Thermo Fisher Scientific, PA1-049), β_2_-AR rabbit polyclonal antibody (1:200, Thermo Fisher Scientific, BS-21452R), and β_3_-AR rabbit polyclonal antibody (1:200, Thermo Fisher Scientific, PA5-143874). CD31/PECAM-1 (R&D Systems, AF3628) was used as an endothelial marker across all wells. Two wells per slide were designated as controls, receiving only antibody diluent.

The following day, slides were equilibrated at room temperature for 30 min, followed by three 5-minute washes with PBS. Secondary antibodies, matched to the host species of the primary antibodies, were applied at a 1:350 dilution and incubated in the dark at room temperature for one hour. The secondary antibodies used were donkey anti-goat-Alexa Fluor 647 (Invitrogen, A21447), in combination with either donkey anti-mouse-Alexa Fluor 555 (Invitrogen, A31570) or donkey anti-rabbit-Alexa Fluor 555 (Invitrogen, A31572). All secondary antibodies were diluted in DAPI stain (Sigma-Aldrich, 10,236,276,001 Roche), which was pre-diluted in PBS (1:10,000) to label the nuclei, as described by Tarnowski et al. [29]. Control slides were incubated with DAPI stain, donkey anti-goat-Alexa Fluor 647, and either donkey anti-rabbit-Alexa Fluor 555 or donkey anti-mouse-Alexa Fluor 555. Afterward, the slides were washed three times for 5 min each with PBS.

Slides were mounted using Fluoromount aqueous mounting medium (Sigma-Aldrich, F4680) and stored in a covered container at 4°C until imaging the following day. Imaging was performed using an inverted wide-field microscope with ApoTome, with both 20× water immersion and 63× oil immersion objectives. Images were processed using ZEN (blue edition) software.

### 2.3 RT-qPCR analysis of of ERs and β-ARs in female HCAECs

HCAECs at P9 (14 years), P10 (24 years), and P8 (37 years) were cultured in 12-well plates with four biological replicates per female donor. Upon reaching 80–90% confluence, cells were serum-starved for 24 h by replacing the endothelial cell growth MV2 medium with basal endothelial cell growth medium MV2 (Promocell, C-22226), supplemented with 5% charcoal-stripped Fetal Bovine Serum (FBS, Gibco-Thermo Fisher Scientific, A3382101) and without additional supplements, antibiotics [30], [31]. Following serum starvation, cells were washed with cold PBS, and cell lysis was initiated by adding 350 µL of freshly prepared RNeasy lysis buffer (Qiagen, 74004) containing 10 µL of β-mercaptoethanol per 1 mL of buffer. The cells were scraped off with a rotatable cell scraper, and the resulting lysate was stored at −80°C until RNA isolation.

RNA was isolated using the Qiagen RNeasy Micro Kit (Qiagen, 74004) following the manufacturer’s protocol. The isolated RNA was eluted in 14 µL of nuclease-free water, kept on ice, and quantified using a SYNERGY/HTX multimode reader (Gen5 3.13 software). RNA purity was assessed by measuring absorbance at 260 nm and 280 nm, with a ratio of approximately 2.0 indicating pure RNA. RNA was stored at −80°C for subsequent cDNA synthesis, which was carried out within 24 h using the iScript cDNA Synthesis Kit (Bio-Rad, 1708890). The reaction mixture, consisting of 4 µL of 5x reverse-transcription mix, 1 µL of iScript reverse transcriptase, 500 ng of RNA, and nuclease-free water, was incubated using the following program: 25°C for 5 min, 46°C for 20 min, 95°C for 1 min, followed by a hold at 4°C. cDNA was diluted 1:5 with nuclease-free water, and 100 ng of cDNA per sample was stored at −20°C for RT-qPCR.

The reference genes hTBP and hB2M were used for normalization, while the genes of interest included hESR1, hESR2, hGPER1, hADRβ1, hADRβ2, and hADRβ3. Primers for hADRβ1, hADRβ3, and hESR1 were obtained from Bio-Rad (PrimePCR™ SYBR® Green Assay), while others were obtained from Invitrogen as indicated in Table 1.

**Table 1:** Primer sequences used for RT-qPCR in Female HCAECs.

Gene	Forward primer (5′-3′)	Reverse primer (3′-5′)	Amplicon Size (bp)
hTBP	TGTGCTCACCCACCAACAAT	CTGCTCTGACTTTAGCACCTGT	140
hB2M	ATGAGTATGCCTGCCGTGTG	TGCTTACATGTCTCGATCCCAC	77
hESR1	Not disclosed by manufacturer	Not disclosed by manufacturer	69
hESR2	GCCAAGAAGATTCCCGGCTT	AATTGAGCGCCACATCAGCC	108
hGPER1	GTGCTACTCCCTCATTGTCCG	GATGAAGACGTTCTCCGGCA	142
hADRβ1	Not disclosed by manufacturer	Not disclosed by manufacturer	94
hADRβ2	GGGCATCGTCATGTCTCTC	GACGCTCGAACTTGGCAATG	83
hADRβ3	Not disclosed by manufacturer	Not disclosed by manufacturer	77n

Forward and reverse primer sequences are listed for hTBP, hB2M, hESR2, hGPER1, and hADRβ2, along with the expected amplicon size in base pairs (bp). Sequences for ESR1, ADRβ1, and ADRβ3 were not disclosed by the manufacturer.

RT-qPCR was performed using SsoFast™ EvaGreen® Supermix (Bio-Rad, 1725204). Each 10 µL reaction was prepared on ice under subdued light and consisted of 5 µL of SsoFast™ EvaGreen® Supermix, 0.5 µL of each 1:10 diluted forward and reverse primer (Invitrogen) or 0.5 µL of ready-to-use primer mix (Bio-Rad PrimePCR™ SYBR® Green Assay), and RNase-free water to a final volume of 7 µL. Then, 3 µL of cDNA was added to each well. The 384-well RT-qPCR plates were sealed and centrifuged at 4°C, 2000 *g* for 1 min. PCR amplification was carried out using the following conditions: denaturation at 95°C for 1 min, followed by 45 cycles of denaturation at 95°C for 5 s, and annealing/extension at 60°C for 15 s with plate reads. A melting curve analysis was performed from 65°C to 95°C in 0.5°C increments. No-template controls (NTCs) were included on each plate.

Following RT-qPCR, the presence of a single amplicon was verified by agarose gel electrophoresis. A 4% agarose gel was prepared in 1x TBE buffer, stained with GelRed (Biotium, 41003), and electrophoresis with a GeneRuler 50 bp DNA Ladder (Thermo Fisher Scientific, SM0371) as a size marker. Electrophoresis was performed at 120 V for 1–2 h. Gel images were captured using the FUSION FX VILBER system.

It is worth noting that cell lysates collected from different female donors were stored at −80°C until RNA isolation, cDNA synthesis, and RT-qPCR were conducted in parallel.

RT-qPCR data were analyzed using Bio-Rad CFX Maestro 1.1. Ct values and melting curves were assessed for technical duplicates; variations ≤0.3 (Ct 9–30) and ≤1.5 (Ct 31–42) were accepted. Samples with multiple melting peaks were excluded [32]. Mean Ct values were calculated, and ΔCt values were obtained using the geometric mean of hTBP and hB2M reference genes [33]. Relative expression was reported as 2^−ΔCt^ for basal studies and 2^−ΔΔCt^ for stimulation studies [34].

Statistical analysis was performed using GraphPad Prism 11. Basal data were analyzed by two-way ANOVA (mean ± SD), to assess the effects of receptor subtype and donor, as these experiments involved multiple receptor types across different donors. Stimulated conditions were analyzed with Mann-Whitney tests and Holm-Šídák correction (median ± IQR), based on statistical consultation, as the data did not meet the assumptions required for parametric testing. Significance: *p* ≤ 0.05 (*), p ≤ 0.01 (**), p ≤ 0.0001 (***) [35], [36], [37].

### 2.4 Western blot assessment of ERs and β-ARs in female HCAECs

HCAECs at the same passage as used in the IF study were cultured in 6-well plates for protein expression analysis of ERs and β-ARs. Cryopreserved cells from different female donors were thawed and seeded simultaneously, with three biological replicates per donor. Upon reaching 80–90% confluence, cells were serum-starved for 24 h following the protocol of the gene expression study.

Protein extraction was performed using RIPA lysis buffer (Santa Cruz, sc-24948) supplemented with Phenylmethylsulfonyl fluoride (PMSF), protease inhibitors, sodium orthovanadate, and Benzonase® Nuclease (Sigma-Aldrich, E1014-5KU). Lysates were incubated on ice for 30 min, centrifuged (12,000 rpm, 20 min, 4°C), and stored at −80°C. Protein concentration was determined using the Pierce™ BCA kit (Thermo Fisher Scientific, 23227), with quantification via a four-parameter logistic model.

Samples were mixed with 1x Laemmli buffer, phosphatase inhibitors (PhosSTOP), and Dithiothreitol (DTT), then heated (95°C, 5 min). Equal protein amounts (10 µg in 20 µL) were loaded onto 10% SDS-PAGE gels (Bio-Rad Mini-PROTEAN system), with duplicate gels prepared for detection of phosphorylated and total proteins. Spectra™ multicolor ladder (3 µL, Thermo Fisher, 26634) was included in electrophoresis. Electrophoresis was performed initially at 80 V for stacking and then at 120 V for protein resolution.

Proteins were transferred to 0.2-µm nitrocellulose membranes at 25 V and 1 mA for 30 min using the Bio-Rad Trans-Blot Turbo System. Transfer efficiency was verified by Ponceau S staining [38]. Membranes were subsequently blocked with 5% milk in TBST for 1 h at room temperature and incubated overnight at 4°C with primary antibodies: ERα (mouse, Thermo Fisher Scientific, MA1-310, 1:250), ERβ (rabbit, Thermo Fisher Scientific, PA1-311, 1:1000), GPR30 (rabbit, Thermo Fisher Scientific, PA5-28647, 1:1000), and β_2_-AR (rabbit, Abcam, ab182136, 1:1000). Western blot analysis showed multiple non-specific bands for β_1_- and β_3_-AR, therefore they were excluded from the study. Loading controls included anti-vinculin (rabbit, 1:1000; Cell Signaling Technology, 4650), anti-GAPDH (mouse, 1:10,000; Abcam, ab8245), and anti-α-tubulin (mouse, 1:4000; Sigma-Aldrich, T5168). All antibodies were diluted in 5% BSA, except for ERα, GAPDH, α-tubulin, and vinculin, which were prepared in 5% milk.

After overnight incubation with primary antibodies, membranes were washed three times for 10 min each with 1× TBST at room temperature with gentle shaking (20 rpm) to remove unbound antibodies. Membranes were then incubated with species-specific secondary antibodies: peroxidase-conjugated goat anti-rabbit IgG (H + L) (Dianova, 111035003) or goat anti-mouse IgG (Jackson ImmunoResearch, 115035003), diluted 1:2500 in 5% milk, for 1 h at room temperature with gentle shaking (20 rpm).

Following secondary antibody incubation, membranes were washed three times for 10 min each with 1× TBST. TBST was then replaced with TBS to prevent potential interference of Tween-20 with the chemiluminescent signal. Detection was performed using SuperSignal West Pico Chemiluminescent Substrate (Thermo Fisher Scientific, 34580), and membranes were imaged using a FUSION FX VILBER system.

Band intensities were quantified in Image J and normalized to the geometric mean of GAPDH, α-tubulin, and vinculin reference proteins. Statistical analyses were performed using two-way ANOVA, to assess the effects of receptor subtype and donor, as these experiments involved multiple receptor types across different donors. However, β_2_-AR protein expression was analyzed using one-way ANOVA (GraphPad Prism 11), which involved a single receptor type across different donors, because antibodies for β_1_- and β_3_-AR produced multiple non-specific bands, precluding accurate detection. Results are expressed as mean ± SD, with significance set at *p* ≤ 0.05 (*), p ≤ 0.01 (**), p ≤ 0.001 (***) [36], [37].

### 2.5 Stimulation of female HCAECs with estrogen or ER agonists

Cells at the same passage number as those used in the basal mRNA expression studies were washed once with PBS after being serum starved as mentioned in the afformentioned sections and subsequently cultured in basal MV2 phenol red-free medium supplemented with 5% charcoal-stripped FBS. The medium was further supplemented with either 17β-estradiol or selective ER agonists for ERα (PPT), ERβ (DPN), or GPER (G1), as previously described [39], [40], [41]. Stock solutions of 17β-estradiol and the ER agonists were prepared at 10 mM in DMSO and filtered through a 0.22-μm syringe filter. Working solutions of 17β-estradiol were generated by serial dilution in DMSO to achieve final concentrations of 1 μM and 10 μM, which were then further diluted in culture media to reach final concentrations of 1 nM and 10 nM. ER agonists were prepared as 10 μM working solutions and subsequently diluted 1:10 in media to reach a final concentration of 10 nM. The final concentration of DMSO was consistently maintained at 0.1% across all experimental conditions. Control treatments consisted of MV2 phenol red-free medium containing 5% charcoal-stripped FBS and 0.1% DMSO.

Cells were incubated with these treatments for 4, 12, or 24 h. For the 17β-estradiol treatments, four biological replicates were performed, while three biological replicates were included for each ER agonist condition. Following the stimulation period, the mRNA expression levels of β-ARs were assessed using the same RT-qPCR protocols as described for the basal mRNA expression analysis.

### 2.6 Assessment of coeficient variance among donors

To quantify variability in mRNA and protein expressions among donors, both analytical (technical within-donor) and biological (natural inter-donors) coefficients of variation (CV) were calculated for each ER and β-AR subtype according to Flatland et al. [42] and Andersen et al. [43] using the following formulas:

a) Analytical CV per all donors:

$$CV_{\mathrm{analytical}\;\left(\%\right)}\;=\frac{\mathrm{pooled}\;within\;donor\;SD}{\begin{array}{c} \begin{array}{c} \mathrm{Mean}\;all\;donor^{\prime }s\;measurements \end{array} \end{array}}=\frac{\begin{array}{c} \sqrt{\Sigma \;\left(\mathrm{SD}j^{2}\right)\times \frac{\left(\mathrm{nj}\;-\;1\right)}{\Sigma \;\left(\mathrm{nj}\;-\;1\right)}} \end{array}}{\mu \;pooled}$$

∑: summation operator

SDj: standard deviation of replicate measurements for donor j.

nj: number of replicates for donor j.

μ: mean of all measurements across all donors.

b) Total CV per all donors (overll variance):

$$CV_{\mathrm{total}\;\left(\%\right)}=\frac{\begin{array}{c} \mathrm{SD}\;all\;donor^{\prime }s\;measurements \end{array}\;}{\mathrm{Mean}\;all\;donor^{\prime }s\;measurements}$$

c) Biological CV per all donors:

$$CV_{\mathrm{biological}\;\left(\%\right)}=\begin{array}{c} \sqrt{CV_{\mathrm{total}}^{2\;}-CV_{\mathrm{analytical}}^{2\;}} \end{array}$$

## 3 Results

### 3.1 Detection of ERs and β-ARs in female human coronary artery endothelial cells

**Fig. 1:**
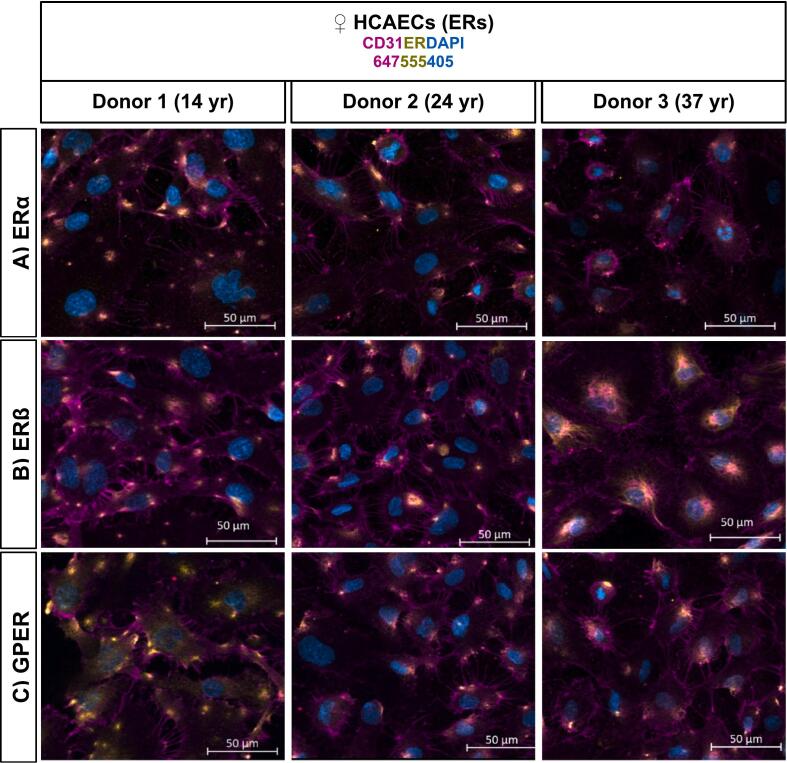
ERs Localization in Female HCAECs. Merged images show ERα (Panel A), ERβ (Panel B), and GPER (Panel C) expression in non-permeabilized HCAECs. Endothelial cells were identified by CD31 staining (Alexa Fluor 647, magenta), nuclei by DAPI (blue), and ERs by Alexa Fluor 555 (yellow). Images were captured using an ApoTome-equipped inverted wide-field microscope (20 ×).

**Fig. 2:**
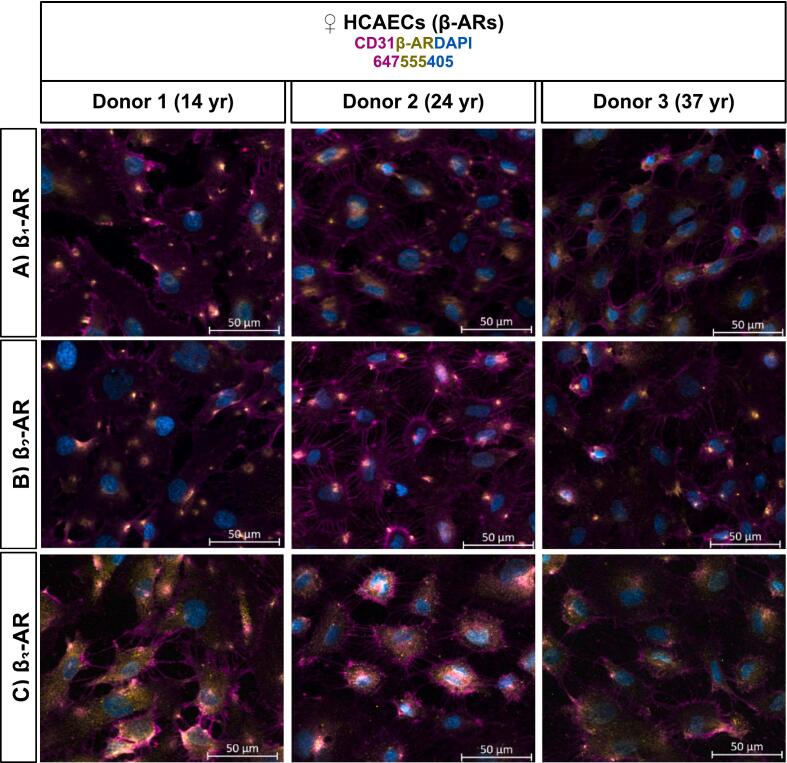
β-AR Localization in Female HCAECs. Merged images show β_1_-AR (Panel A), β_2_-AR (Panel B), and β_3_-AR (Panel C) expression in non-permeabilized HCAECs. Endothelial cells were identified by CD31 staining (Alexa Fluor 647, magenta), nuclei by DAPI (blue), and β-ARs by Alexa Fluor 555 (yellow). Images were captured using an ApoTome-equipped inverted wide-field microscope (20×).

**Fig. 3:**
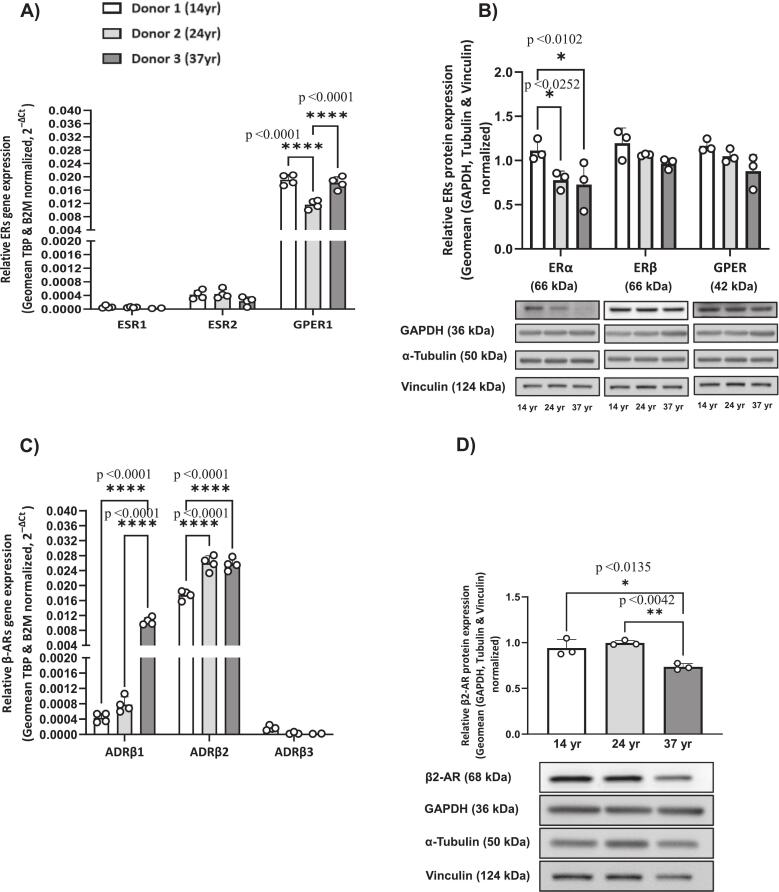
Basal Expression of ERs and β-ARs in Female HCAECs. Basal mRNA and protein levels of ERs (ERα, ERβ, GPER1) and β-ARs (β_1_-AR, β_2_-AR, β_3_-AR) in HCAECs from female donors (14, 24, 37 yrs). Panels A–B: ER mRNA/protein expression; C–D: β-AR mRNA expression and β2-AR protein. Data are presented as mean ± SD. ERβ and β_2_-AR bands observed at ∼66 and ∼68 kDa, respectively.

**Fig. 4:**
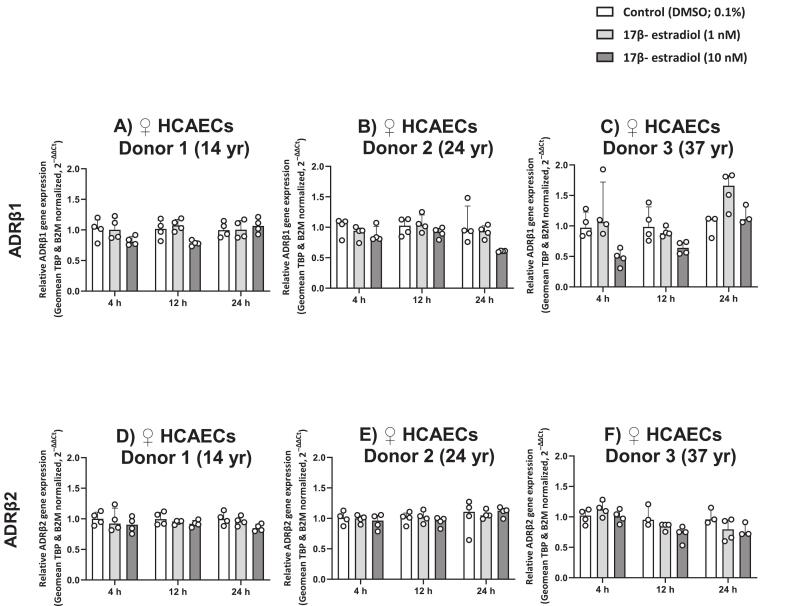
17β-Estradiol does not affect β-AR mRNA Expression in Female HCAECs. mRNA expression of ADRβ1 (A–C), and ADRβ2 (D–F) was analyzed in HCAECs from female donors aged 14, 24, and 37 years following 17β-estradiol stimulation (1 nM or 10 nM) for 4, 12, or 24 h, compared to vehicle control (0.1% DMSO). Data was obtained from four biological replicates per condition per donor (n = 4) and are presented as median with IQR.

**Fig. 5:**
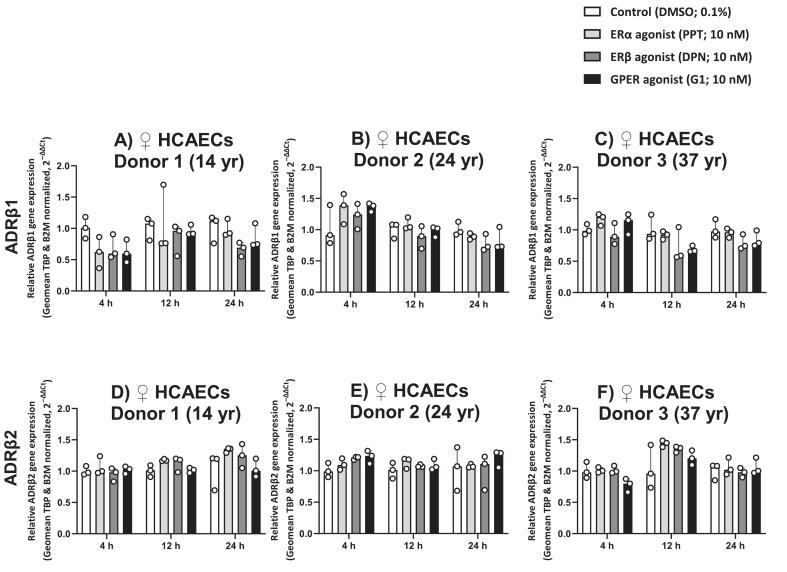
ER Agonists do not modulate β-AR Gene Expression in Female HCAECs. Gene expression of ADRβ1 (A–C) and ADRβ2 (D–F) was assessed in HCAECs from female donors aged 14, 24, and 37 years following stimulation with 10 nM of ERα (PPT), ERβ (DPN), or GPER (G1) agonists for 4, 12, or 24 h, compared to 0.1% DMSO control. Data from three biological replicates per condition per donor (n = 3) are shown as median with IQR.

IF staining indicated the expression of ER subtypes—ERα (Fig. 1A), ERβ (Fig. 1B), and GPER (Fig. 1C)—as well as β-AR subtypes—β_1_-AR (Fig. 2A), β_2_-AR (Fig. 2B), and β_3_-AR (Fig. 2C)—in HCAECs from three healthy female donors aged 14, 24, and 37 years. Additional images, including 63× non-permeabilized, 20× and 63× permeabilized, and control images, are provided in Appendix, Figs. 1–8.

### 3.2 Differential expression of estrogen and β-adrenergic receptors in female HCAECs from diverse donors

mRNA expression of ERs analyzed by two-way ANOVA revealed significant main effects of receptor subtype and donor, as well as a significant interaction between these factors (p < 0.001), indicating that receptor expression varies across donors and that differences between receptor subtypes are donor dependent. Overall, ERα exhibited the lowest expression, whereas GPER was the most abundantly expressed among the ER subtypes in female HCAECs (Fig. 3A). Post hoc Tukey multiple comparisons further identified specific differences between donors. Notably, GPER expression varied among donors, with a biological CV of 20.8% (Table 2), and higher levels were observed in cells from donor 1 (14 yr) and donor 3 (37 yr) compared to those from donor 2 (24 yr). Protein expressions analysed by two-way ANOVA revealed significant main effects of receptor subtype (p < 0.05) and donor (p < 0.001), with no significant interaction between these factors, indicating that differences in receptor expression are consistent across donors despite overall inter-donor variability. Overall, western blot indicated that ERβ and GPER had slightly higher basal protein levels than ERα (Fig. 3B). Post hoc Tukey multiple comparisons further identified specific differences between donors. Notably, ERα protein expression also differed across donors, with the highest levels detected in cells from donor 1 (14 yr), with biological CVs of 17.8% (Table 2).

**Table 2:** Coefficients of variation of ER and β-AR mRNA and protein expression across female HCAEC donors.

	CV pooled analytical (%)	CV total (%)	CV biological (%)
ERα mRNA	53.6519821	54.72653573	10.79159533
ERα protein	21.13896976	27.65084008	17.82450321
ERβ mRNA	35.3527142	42.01498322	22.70340094
ERβ protein	9.80256339	12.60248443	7.920376558
GPER mRNA	9.868272881	23.06156903	20.84353992
GPER protein	11.93959495	15.93430846	10.55216843
β_1_-AR mRNA	14.94277228	125.3901844	124.4966341
β_2_-AR mRNA	7.096509862	19.05538163	17.68465767
β_2_-AR protein	6.720570283	14.60373686	12.96545662
β_3_-AR mRNA	49.27871954	92.1898323	77.91388182

Pooled analytical, total, and biological CVs are shown for ERα, ERβ, and GPER mRNA and proteins, for β_1_-AR, β_2_-AR, and β_3_-AR mRNA, and for β_2_-AR protein only. All values are expressed as percentages (%).

Regarding mRNA expression of β-ARs analyzed by two-way ANOVA revealed significant main effects of receptor subtype and donor, as well as a significant interaction between these factors (p < 0.001), indicating that receptor expression varies across donors and that differences between receptor subtypes are donor-dependent. Overall, β_3_-AR was the least expressed subtype, while β_2_-AR was the most highly expressed (Fig. 3C). Post hoc Tukey multiple comparisons further identified specific differences between donors. Notably, donor-specific differences were evident in β_1_-AR and β_2_-AR mRNA expression. β_1_-AR levels were higher in cells from donor 3 (37 yr) compared to those from donor 1 (14 yr) and donor 2 (24 yr), whereas β_2_-AR expression was elevated in cells from donor 2 (24 yr) and donor 3 (37 yr) compared to those from donor 1 (14 yr). At the protein level, β_2_-AR expression analyzed by one-way ANOVA was analyzed using one-way ANOVA, which revealed a significant effect of donor (p < 0.01), indicating inter-donor variability. Post hoc Tukey multiple comparisons further identified specific differences between donors, β_2_-AR expression was lower in cells from donor 3 (37 yr) compared to those from donor 1 (14 yr) and donor 2 (24 yr) (Fig. 3D). Protein-level analysis was restricted to β_2_-AR because specific and reliable antibodies for β_1_- and β_3_-ARs were not available, antibodies for β_1_- and β_3_-AR produced multiple non-specific bands, precluding accurate detection.

Biological variability was observed for β-ARs, with β_2_-AR mRNA and protein exhibiting biological CVs of 17.6% and 12.9%, respectively, while β_1_-AR mRNA displayed markedly higher biological variability, with CVs of 124.4% (Table 2). Interestingly, the mRNA expression levels of ERs and β-ARs did not reflect the corresponding protein expression profiles (Fig. 3A-D). Corresponding agarose gel images for the mRNA analyses can be found in Appendix, Fig. 9.

### 3.3 Correlation between 17β-estradiol and β-adrenergic receptor gene expression in female HCAECs

Stimulation of female HCAECs with 17β-estradiol at concentrations of 1 nM or 10 nM for 4, 12, or 24 h did not produce significant changes in mRNA expression levels of any β-AR subtypes. Specifically, no significant changes were detected in the expression of β_1_-AR (Fig. 4A–C), and β_2_-AR (Fig. 4D–F) across HCAECs from all donors. For β_3_-AR, although a RT-qPCR-amplified fragment was detectable on agarose gel (Appendix, Fig. 9C), its expression levels were very low and below the reliable detection threshold in quantitative analysis; therefore, it was not included in further analyses.

### 3.4 Correlation between estrogen receptor activation and β-adrenergic receptor expression in female HCAECs

Stimulation of female HCAECs with selective ER agonists (10 nM) for 4, 12, or 24 h did not induce significant changes in the mRNA expression levels of β-AR subtypes. This included β_1_-AR (Fig. 5A–C), and β_2_-AR (Fig. 5D–F) across HCAECs from all donors. For β_3_-AR, although a RT-qPCR-amplified fragment was detectable on agarose gel (Appendix, Fig. 9C), its expression levels were very low and below the reliable detection threshold in quantitative analysis; therefore, it was not included in further analyses.

## 4 Discussion

Sex-specific vascular differences, particularly around menopause, are largely influenced by female sex hormones [14]. Although estrogen exerts well-documented vascular benefits, its role in modulating cardiovascular risk remains incompletely understood [3]. Emerging evidence indicates that estrogen may interact with β-ARs, contributing to sex-specific differences in sympathetic nervous system activity and vascular responsiveness [2], [44], [45]. However, the mechanisms underlying ER–β-AR cross-talk in HCAECs are poorly defined. Most previous studies have relied on non-coronary endothelial cells or animal models, which may not accurately replicate the signaling environment of the human coronary endothelium. Given that ischemic heart disease in women is frequently associated with microvascular dysfunction and coronary tone abnormalities [3], [4], we employed HCAECs derived from three young, healthy female donors whose deaths were accidental and who had no recorded cardiovascular disease or other comorbidities. This model enabled characterization of ER and β-AR signaling while minimizing confounding effects from disease-related endothelial alterations, providing a clearer basis for understanding sex-specific expression patterns.

In this study, the basal expression of ERα, ERβ, GPER, β_1_-, β_2_-, and β_3_-ARs was examined at both the mRNA and protein levels using RT-qPCR, IF staining, and western blotting, with calculation of inter-donor variability (biological CVs). HCAECs were further stimulated with 17β-estradiol (1 or 10 nM) or selective ER agonists (10 nM) for 4, 12, or 24 h to assess potential effects on β-AR gene expression. The results demonstrated the presence of all ER and β-AR subtypes in HCAECs from all donors, with notable inter-donor variability in gene and protein expression profiles. Stimulation with 17β-estradiol or selective ER agonists in the experimental setting did not significantly alter β-AR gene expression. These findings emphasize the need to consider biological variability in human endothelial signaling studies, which may contribute to the heterogeneous cardiovascular responses to hormonal and adrenergic stimuli observed clinically.

IF analysis confirmed that both ER subtypes (ERα, ERβ, GPER) and β-AR subtypes (β_1_-, β_2_-, β_3_-AR) are expressed in HCAECs from three female donors aged 14, 24, and 37 years, with both intracellular and membrane-associated localization observed (Fig. 1, Fig. 2; Appendix, Figs. 1–8). This dual localization suggests that these receptors are positioned to mediate both genomic and non-genomic signaling, and their co-expression supports the potential for complex cross-talk between estrogen- and adrenergic-mediated pathways in coronary endothelial cells.

Among ER subtypes, ERα exhibited the lowest basal expression, whereas GPER was the most abundantly expressed (Fig. 3A). This observation aligns with previous research by Ghaffari et al. [46], who reported a similar expression pattern in HCAECs from both male and female donors aged 27 to 63 yr. GPER mRNA expression varied significantly among donors, with a biological CV of 20.8%, while GPER showed relatively low variability at the protein level (CVs 10.5%) as indicated in Fig. 3A, B; Table 2. ERα protein levels differed significantly between donors, with the highest expression in cells from donor 1 (14 yr). For β-ARs, β_2_-AR was the predominant subtype at mRNA level, whereas β_3_-AR expression was minimal (Fig. 3C, D). Donor-specific differences were apparent for β_1_-AR and β_2_-AR, with β_2_-AR mRNA and protein showing moderate biological variability (CVs 17.6% and 12.9%, respectively), while β_1_-AR mRNA displayed markedly higher biological variability (CVs 124.4%; Table 2). Although β_1_- and β_3_-AR antibodies produced distinct staining in IF, western blot analysis showed multiple non-specific bands. Therefore, β_1_- and β_3_-AR were not included in the quantitative protein analysis.

These observations highlight that human coronary endothelial receptor expression is subject to substantial inter-donor variability, potentially influenced by donor age, biological background, and cell source or processing conditions, which may influence individual differences in endothelial responsiveness to hormonal and adrenergic stimuli. Furthermore, the co-expression of ERs and β-ARs at both membrane and intracellular locations suggests a structural basis for potential ER–β-AR cross-talk, which could contribute to sex-specific vascular regulation. Due to inter-donor variability in basal receptor expression, HCAECs from each donor were stimulated and analyzed individually.

Stimulation with 17β-estradiol (1 or 10 nM) or selective ER agonists (PPT, DPN, or G1, 10 nM) for 4 h, 12 h, or 24 h did not significantly alter β_1_-AR or β_2_-AR expression (Fig. 4, Fig. 5). Notably, β_3_-AR transcripts were visible as a RT-qPCR product on agarose gel (Appendix, Fig. 9), but their levels were too low for reliable quantification and were thus excluded from subsequent analyses. It is important to note that these stimulation experiments were conducted as a secondary, exploratory assessment aimed at determining the feasibility of detecting transcriptional changes in β-ARs under the tested conditions, and should be interpreted with caution given the limited exposure duration, concentration, and inter-donor variability.

These results contrast with earlier studies conducted in OVX female Wistar rats, where estrogen supplementation was shown to influence the expression of β-AR subtypes. For example, Riedel et al. [23] reported a decrease in mRNA levels of β_1_-AR and β_3_-AR in the thoracic aorta of OVX rats, which was reversed after biweekly oral estrogen supplementation (2 mg/kg body weight) over seven weeks. Tazumi et al. [25] also observed a significant increase in β_2_-AR mRNA expression in the mesenteric arteries of OVX rats following 90 days of sustained estrogen release (2.5 mg), compared to placebo-treated animals. Yamaguchi et al. [47] found that subcutaneous administration of 17β-estradiol (50 mg/kg) for three days reduced isoprenaline-induced relaxation in aortic rings of OVX rats. In the context of cardiac tissues, Thawornkaiwong et al. [48] demonstrated that β_1_-AR density increased in the hearts of OVX rats, an effect that was attenuated by subcutaneous estrogen injections (5 µg/rat) administered three times per week over a 10-week period. Wu et al. [18] also observed upregulation of β_1_-AR expression and downregulation of β_2_-AR expression in the hearts of OVX rats subjected to ischemia–reperfusion injury, with both expression patterns returning to baseline after 17β-estradiol treatment (40 μg/kg in 100 μL olive oil, subcutaneously administered) over four weeks. Kang et al. [49] found that estrogen treatment via a minipump (40 mg/kg/day for two weeks) normalized β_1_-AR expression and increased β_2_-AR expression in female rats, with these effects mediated through GPER. In non-cardiac tissues like brown adipose tissue, Malo and Puerta [50] reported an increase in β_3_-AR expression in rats following the implantation of silastic capsules containing 17β-estradiol (5 mg over two weeks). Conversely, Monjo et al. [51] observed a decrease in β_3_-AR expression in murine 3 T3-L1 preadipocytes treated with 17β-estradiol at concentrations ranging from 10⁻^9^ to 10⁻^7^ M for 48 h.

These varying findings highlight the intricate and context-dependent nature of estrogen’s regulation of β-AR subtypes. The differences observed between the current study and previous research may be due to several factors, including variations in species, tissue types, experimental setups, and estrogen administration protocols. It is also possible that post-transcriptional or signaling-level mechanisms, rather than transcriptional changes, mediate potential ER–β-AR cross-talk in human coronary endothelial cells. Most previous studies examining the effects of estrogen on β-AR expression have been conducted in OVX rats, which received estrogen supplementation over extended periods, often lasting several weeks. Studies in cell culture generally employed exposure durations of around 48 h. In contrast, the present study limited estrogen treatment to a maximum of 24 h, constrained by the need for serum starvation to minimize background hormonal influences while maintaining HCAEC viability.

## 5 Conclusion

This study demonstrates substantial inter-donor variability in the basal mRNA and protein expression of ERs and β-ARs in HCAECs, highlighting a key challenge in interpreting and generalizing results from estrogen or ER agonist stimulation experiments. Our stimulation experiments were exploratory and should be interpreted with caution. Under the conditions tested, 17β-estradiol (1 or 10 nM, 4, 12, or 24 h) did not produce measurable changes in β-AR mRNA expression, suggesting that short-term ER activation does not directly regulate these adrenergic receptor subtypes at the transcriptional level in HCAECs. These findings contrast with some previous reports on rats, reflecting the complex and context-dependent nature of estrogen’s influence on β-AR regulation. Such discrepancies may arise from differences in tissue types, species, experimental models (in vitro vs. *in vivo*), estrogen administration protocols, or the basal expression levels of ER and β-AR subtypes.

## 6 Limitations

This study provides a descriptive analysis of ERs (ERα, ERβ, GPER) and β-ARs (β_1_-AR, β_2_-AR, β_3_-AR) expression in female HCAECs and explores transcriptional responses following receptor activation. The in vitro approach offers advantages by enabling controlled assessment of drug–receptor interactions independent of systemic influences, such as metabolism or pharmacokinetics. However, several limitations should be considered when interpreting the findings.

In vitro models inherently lack the full vascular environment, including interactions with other vessel layers, neurohumoral regulation, and physiological shear stress, which may influence endothelial gene expression and contribute to the absence of detectable effects. As a result, cellular responses observed in vitro may not fully reflect those occurring under physiological conditions [52], [53].

The experimental concentrations and exposure durations (1 or 10 nM 17β-estradiol; 10 nM selective ER agonists; 4, 12, or 24 h) were chosen based on previous literature to capture early-to-intermediate transcriptional responses. One key challenge in estrogen-related in vitro studies is the selection of an appropriate experimental concentration that accurately mimics physiological conditions. While some studies suggest that micromolar concentrations are necessary to induce response [54], [55], others report efficacy at nanomolar levels [56]. Estrogen’s lipophilic nature allows it to accumulate in tissues, potentially reaching concentrations higher than in plasma. However, in vitro conditions lack carrier proteins, such as sex hormone-binding globulin, which can affect its solubility, bioavailability, and cellular uptake [57], [58], [59]. Exposure was limited to 24 h because cells were maintained under serum-starved conditions during stimulation, and prolonged starvation could compromise cell viability or independently alter gene expression. These conditions were selected to balance the capture of dynamic transcriptional changes with the minimization of confounding factors. Nonetheless, shorter exposure times, lower concentration, lower receptor expression, and inter-donor variability may have limited the detection of subtle, delayed, or dose-dependent transcriptional responses.

Functional validation of receptor engagement was not performed, and β-AR regulation was assessed only at the mRNA level. Protein expression, receptor coupling, or downstream signaling pathways were not examined. Protein-level analysis of β_1_- and β_3_-AR was not feasible due to antibody specificity issues, precluding reliable quantification. Consequently, the absence of transcriptional changes does not exclude potential functional regulation by ERs at post-transcriptional or signaling levels.

Finally, the use of primary HCAECs introduces practical limitations, including restricted cell availability, limited proliferative capacity, and challenges in performing multiple experimental conditions per donor. Obtaining healthy, disease-free female HCAECs is particularly difficult due to donor age and prevalence of cardiovascular disease or related risk factors. Biological replicates could not be pooled due to donor variability, further restricting the number of replicates per donor.

Taken together, these limitations indicate that the present findings should be interpreted within the specific experimental context and do not exclude potential roles of ERs in modulating β-AR function.

## 7 Future perspectives

Future studies should incorporate functional validation of ER activation, including assessment of ER-responsive target genes, selective receptor antagonists, and genetic approaches such as receptor knockdown or silencing. Expanding experimental conditions to include multiple ligand concentrations and longer stimulation durations will help clarify dose- and time-dependent effects.

Development or validation of highly specific monoclonal antibodies for β_1_- and β_3_-ARs is essential for reliable protein-level analysis. Complementary investigations of receptor signaling, coupling efficiency, and downstream functional outcomes will further elucidate the role of ERs in modulating β-adrenergic pathways.

Increasing the number of donor samples and examining sex-, age-, or disease-related variability will provide deeper insight into the physiological and clinical relevance of estrogen–β-adrenergic interactions in the human coronary endothelium. Integrating in vitro and in vivo approaches, including co-culture systems, 3D tissue-engineered vessels, or organ-on-a-chip platforms with controlled shear stress, will allow the study of receptor interactions under more physiologically relevant conditions. Employing genetic tools, such as CRISPR-Cas9 knockout or knockdown of specific ERs or β-ARs, may provide clearer mechanistic insights than pharmacological interventions alone.

Together, these strategies will improve the precision of protein-level analyses, enhance understanding of endocrine cross-talk, and advance knowledge of how estrogen modulates vascular function in a specific context.

## CRediT authorship contribution statement

**Basant Elsaid:** Writing – review & editing, Writing – original draft, Visualization, Validation, Project administration, Methodology, Investigation, Funding acquisition, Formal analysis, Data curation, Conceptualization. **Irakli Kopaliani:** Supervision. **Ansam Seif:** Supervision. **Stephan Speier:** Resources. **Andreas Deussen:** Writing – review & editing, Supervision, Conceptualization.

## Funding

This research was supported by institutional funding from the Institute of Physiology at TU Dresden. Basant Elsaid gratefully acknowledges financial support for living expenses from the German Egyptian Research Long-Term Scholarship Program (DAAD-GERLS), the Cultural Affairs and Missions Sector in Egypt, the Association of Friends and Sponsors of TU Dresden (GFF), and the TU Dresden Graduate Academy (Completion and Wrap-up Grant).

## Declaration of competing interest

The authors declare that they have no known competing financial interests or personal relationships that could have appeared to influence the work reported in this paper.

## Data Availability

The datasets generated during and/or analyzed in the current study are available from the corresponding author on reasonable request.
