# Supplementary material for: Heterogeneity of estrogen and β-adrenergic receptors in female human coronary artery endothelial cells
Source: Int J Cardiol Heart Vasc. 2026 May 15;64:101938. doi: 10.1016/j.ijcha.2026.101938 (PMC13199820; doi:10.1016/j.ijcha.2026.101938)
Supplement: Supplementary Data 1 [file mmc1.pdf]

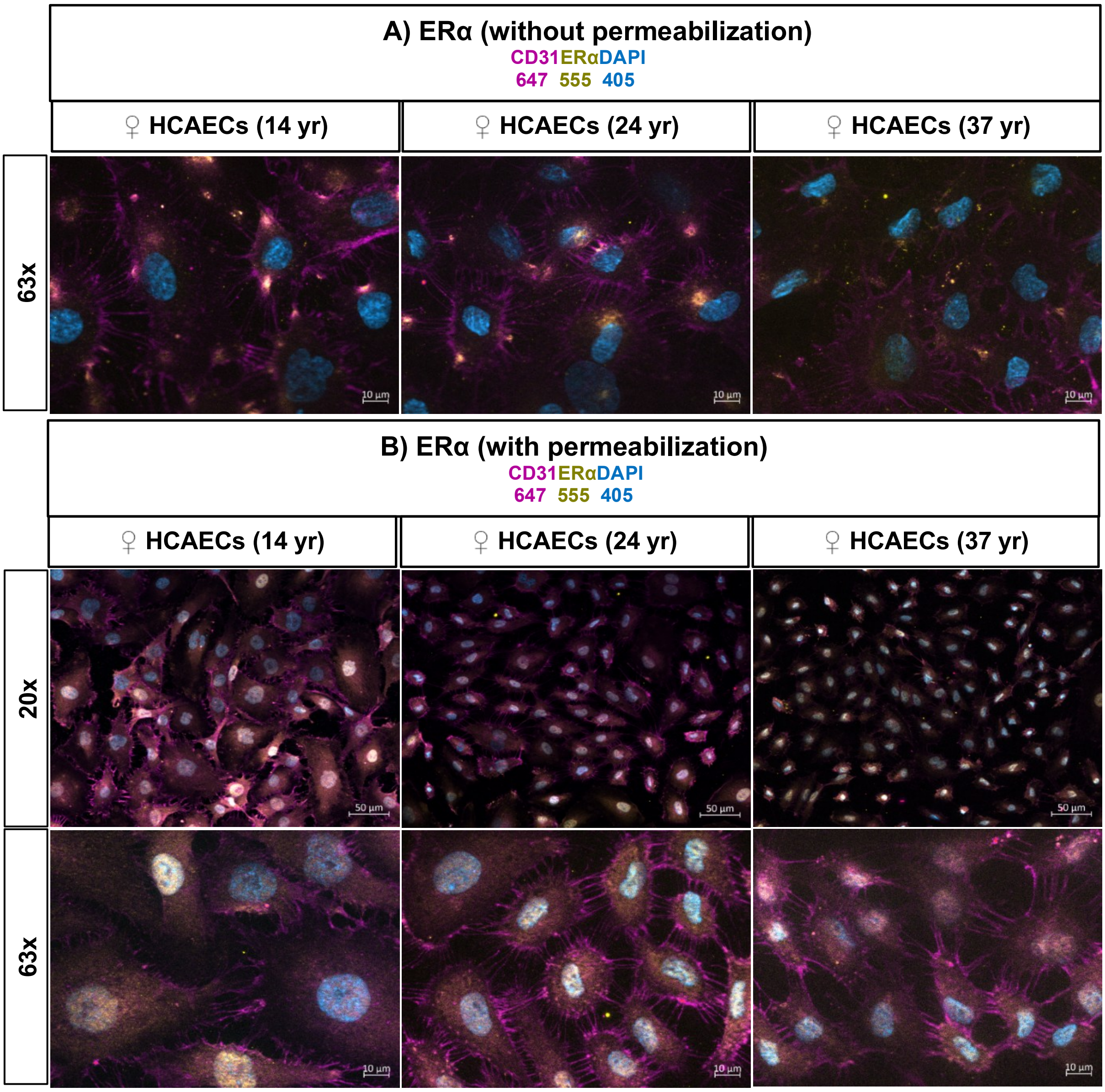

Appendix, Figure 1: ERα Localization in Female HCAECs
Merged immunofluorescence images depict ERα expression in HCAECs from different female donors. Endothelial cells are identified by CD31 staining with Alexa Fluor 647 (magenta), nuclei by DAPI (blue), and ERα by Alexa Fluor 555 (yellow). Panel A shows non-permeabilized samples; Panel B shows permeabilized samples. Imaging was conducted using an inverted wide-field microscope with ApoTome (20× and 63× objectives).

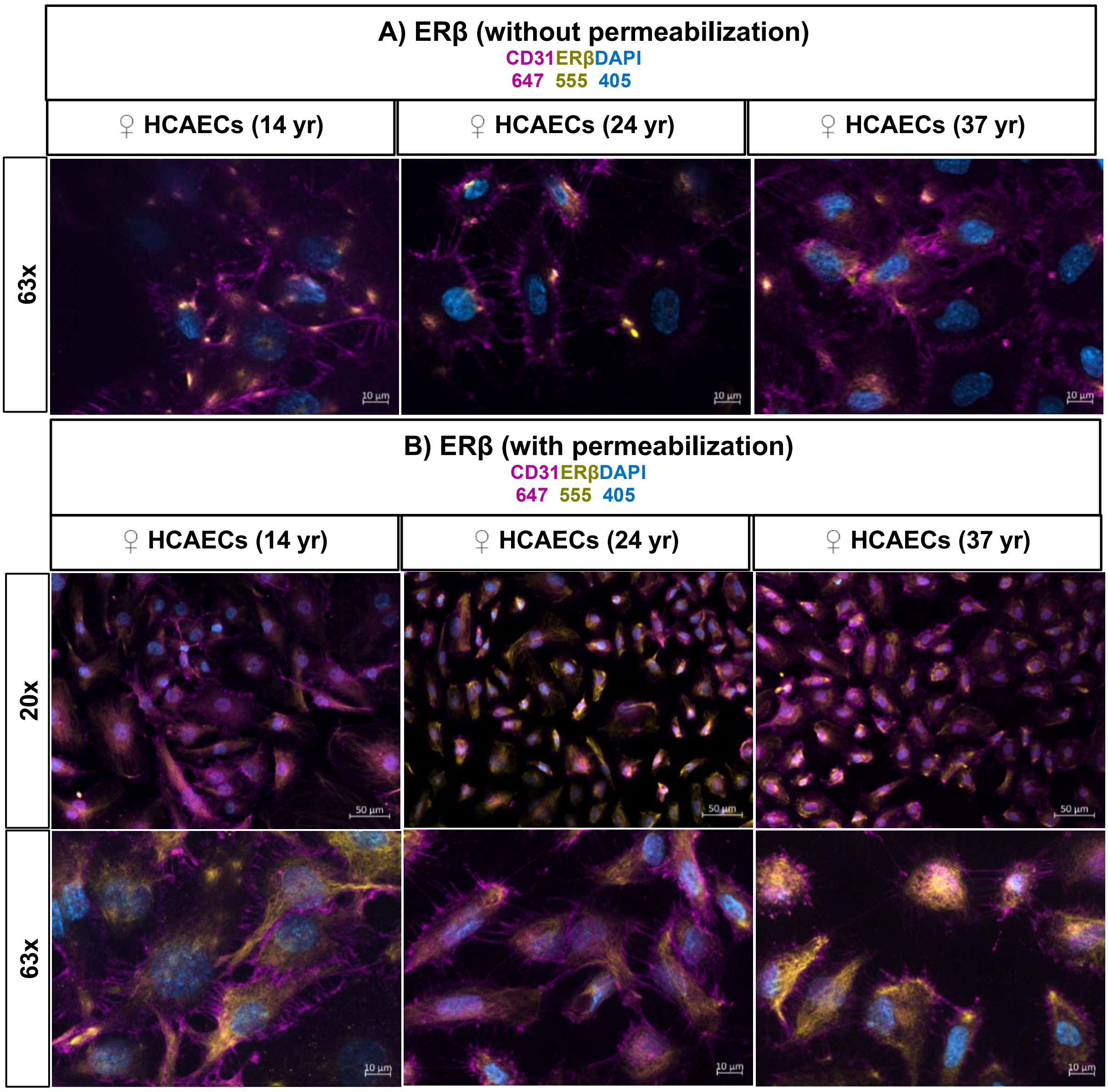

Appendix, Figure 2: ERβ Localization in Female HCAECs
Merged immunofluorescence images illustrate ERβ expression in HCAECs from different female donors. Endothelial cells are labeled with CD31 (Alexa Fluor 647, magenta), nuclei with DAPI (blue), and ERβ with Alexa Fluor 555 (yellow). Panel A shows non-permeabilized samples; Panel B shows permeabilized samples. Imaging was conducted using an inverted wide-field microscope with ApoTome (20× and 63× objectives).

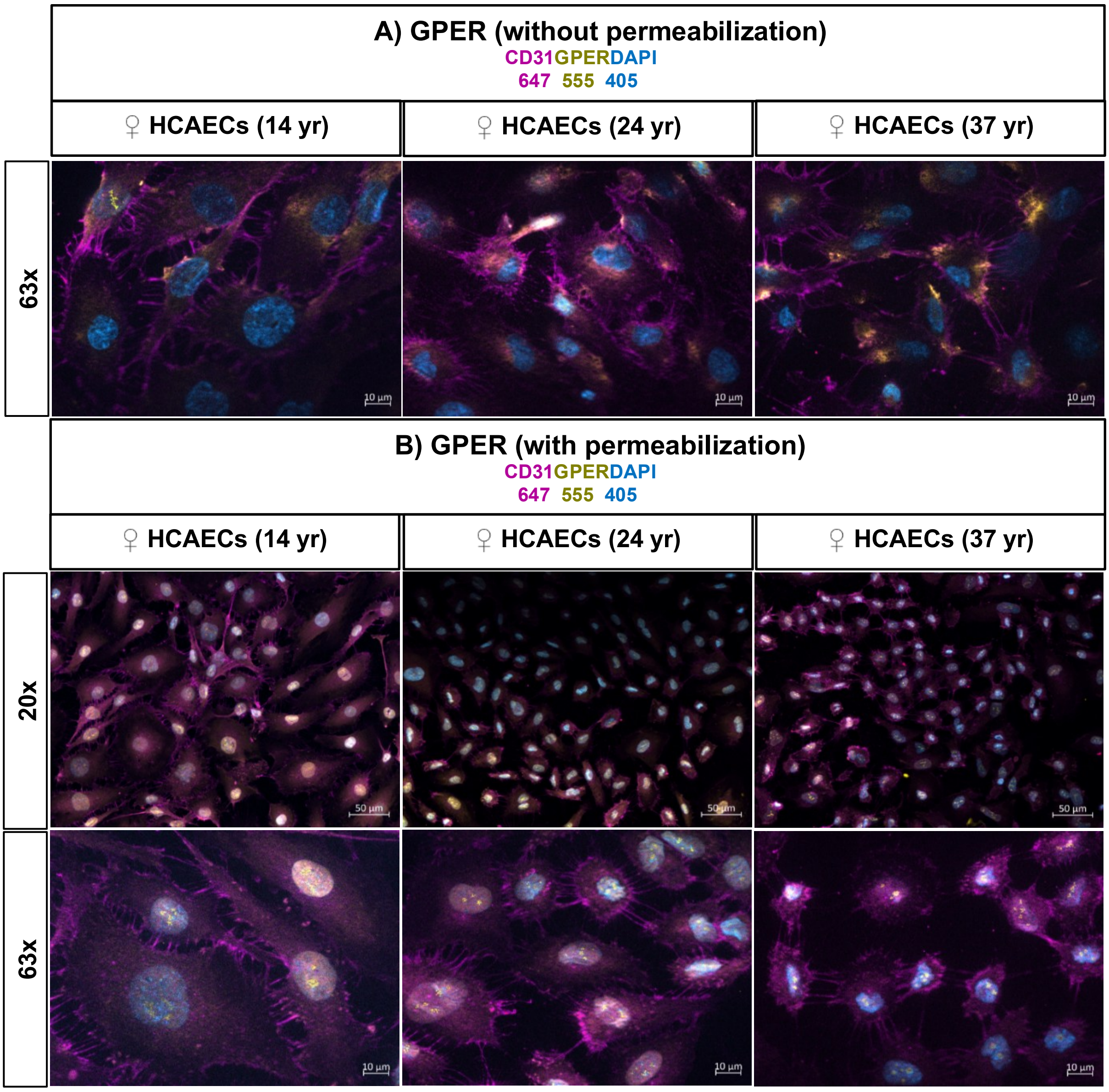

Appendix, Figure 3: GPER Localization in Female HCAECs

Merged immunofluorescence images display GPER expression in HCAECs from different female donors. Endothelial cells are labeled with CD31 (Alexa Fluor 647, magenta), nuclei with DAPI (blue), and GPER with Alexa Fluor 555 (yellow). Panel A shows non-permeabilized samples; Panel B shows permeabilized samples. Imaging was conducted using an inverted wide-field microscope with ApoTome (20× and 63× objectives).

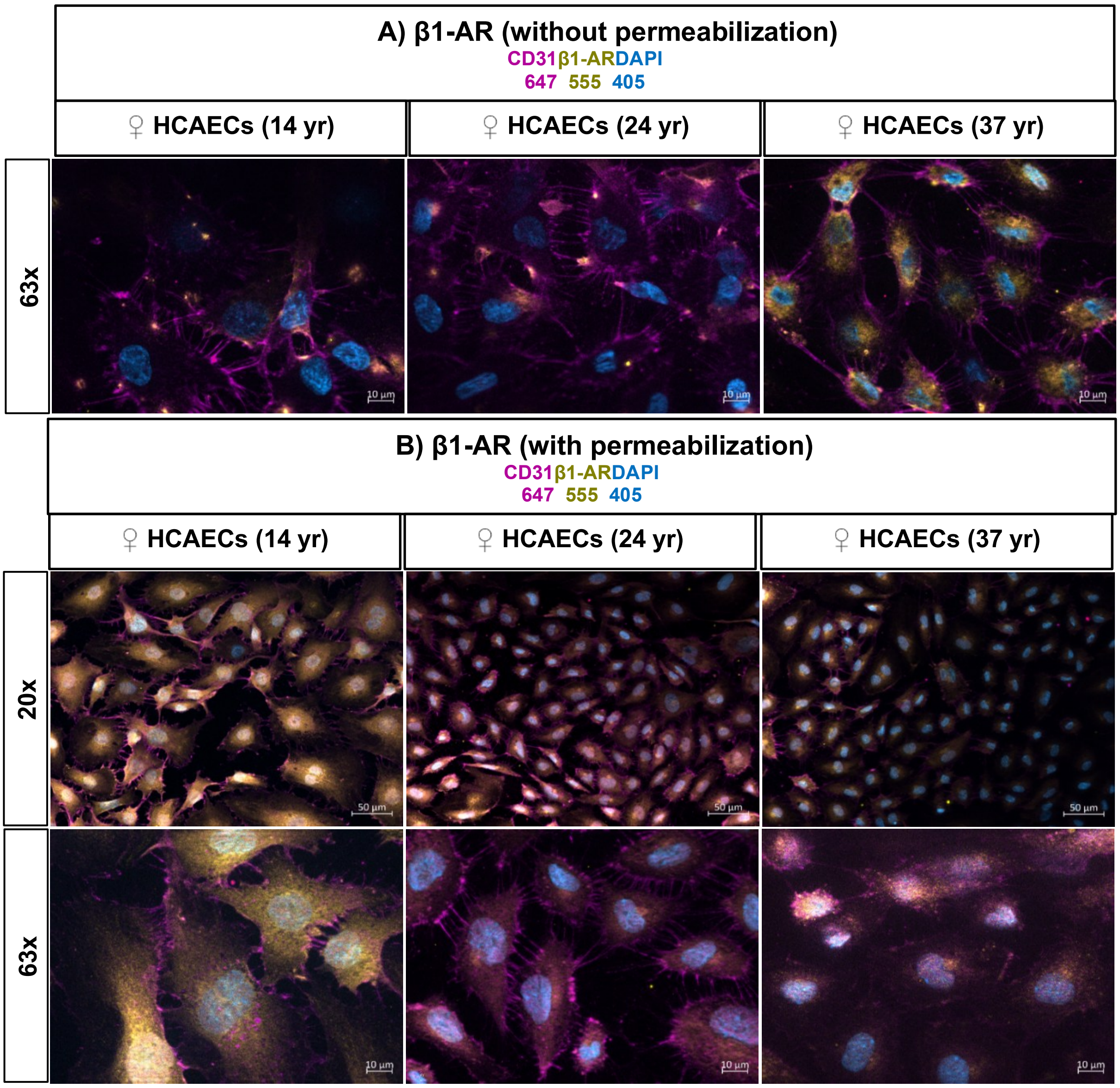

Appendix, Figure 4: β1-Adrenergic Receptor Localization in Female HCAECs
Merged immunofluorescence images depict β1-AR expression in HCAECs from different female donors. Endothelial cells are labeled with CD31 (Alexa Fluor 647, magenta), nuclei with DAPI (blue), and β1-AR with Alexa Fluor 555 (yellow). The experimental design and imaging parameters are consistent with those described in Figure 3.

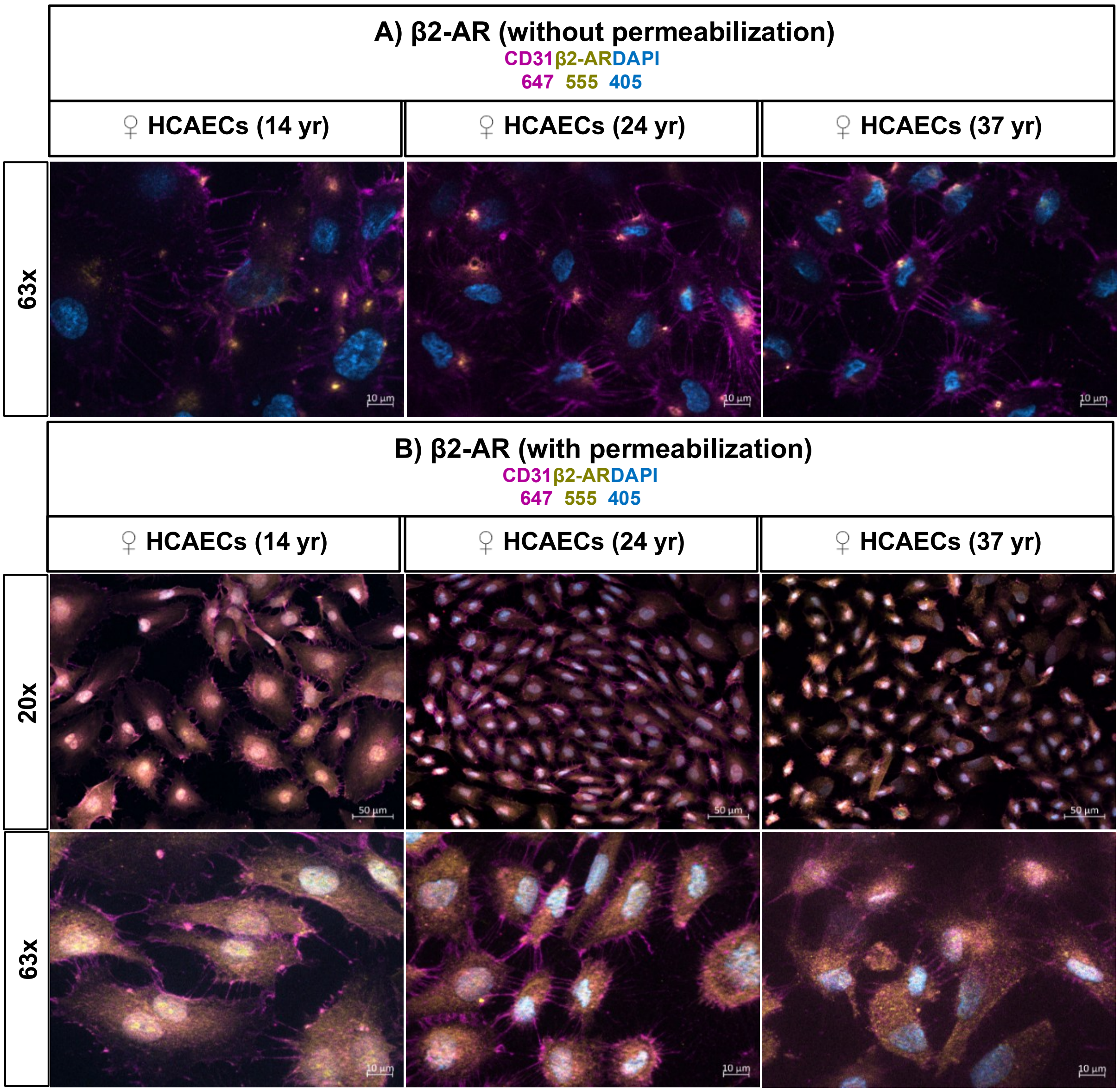

Appendix, Figure 5: β₂-Adrenergic Receptor Localization in Female HCAECs
Merged immunofluorescence images depict β_2-AR expression in HCAECs from different female donors. Endothelial cells are labeled with CD31 (Alexa Fluor 647, magenta), nuclei with DAPI (blue), and β_2-AR with Alexa Fluor 555 (yellow). The experimental design and imaging parameters are consistent with those described in Figure 3.

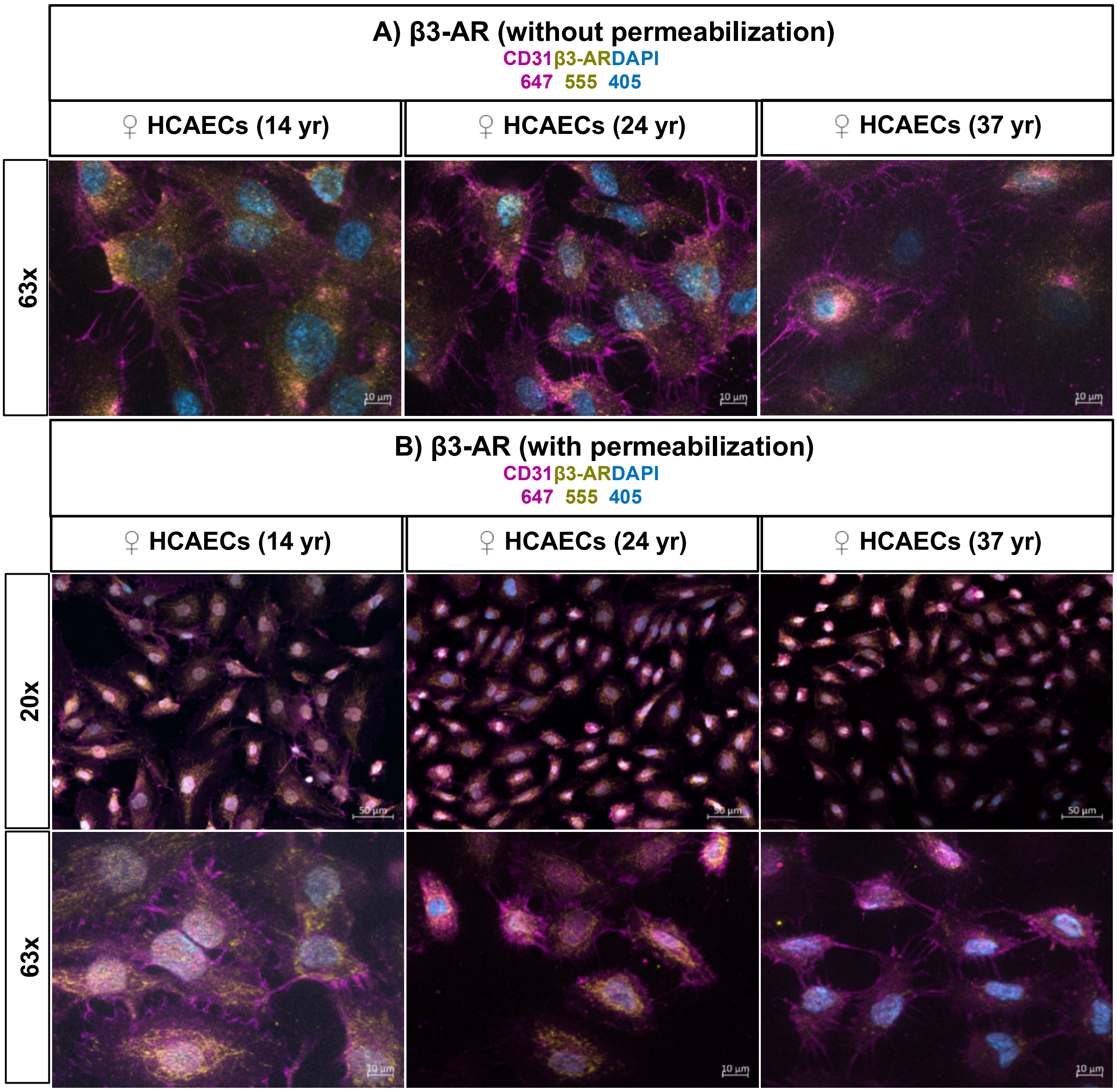

Appendix, Figure 6: $β_3$-Adrenergic Receptor Localization in Female HCAECs
Merged immunofluorescence images illustrate $β_3$-AR expression in HCAECs derived from different female donors. Endothelial cells are labeled with CD31 (Alexa Fluor 647, magenta), nuclei with DAPI (blue), and $β_3$-AR with Alexa Fluor 555 (yellow). The experimental design and imaging parameters are consistent with those described in Figure 3.

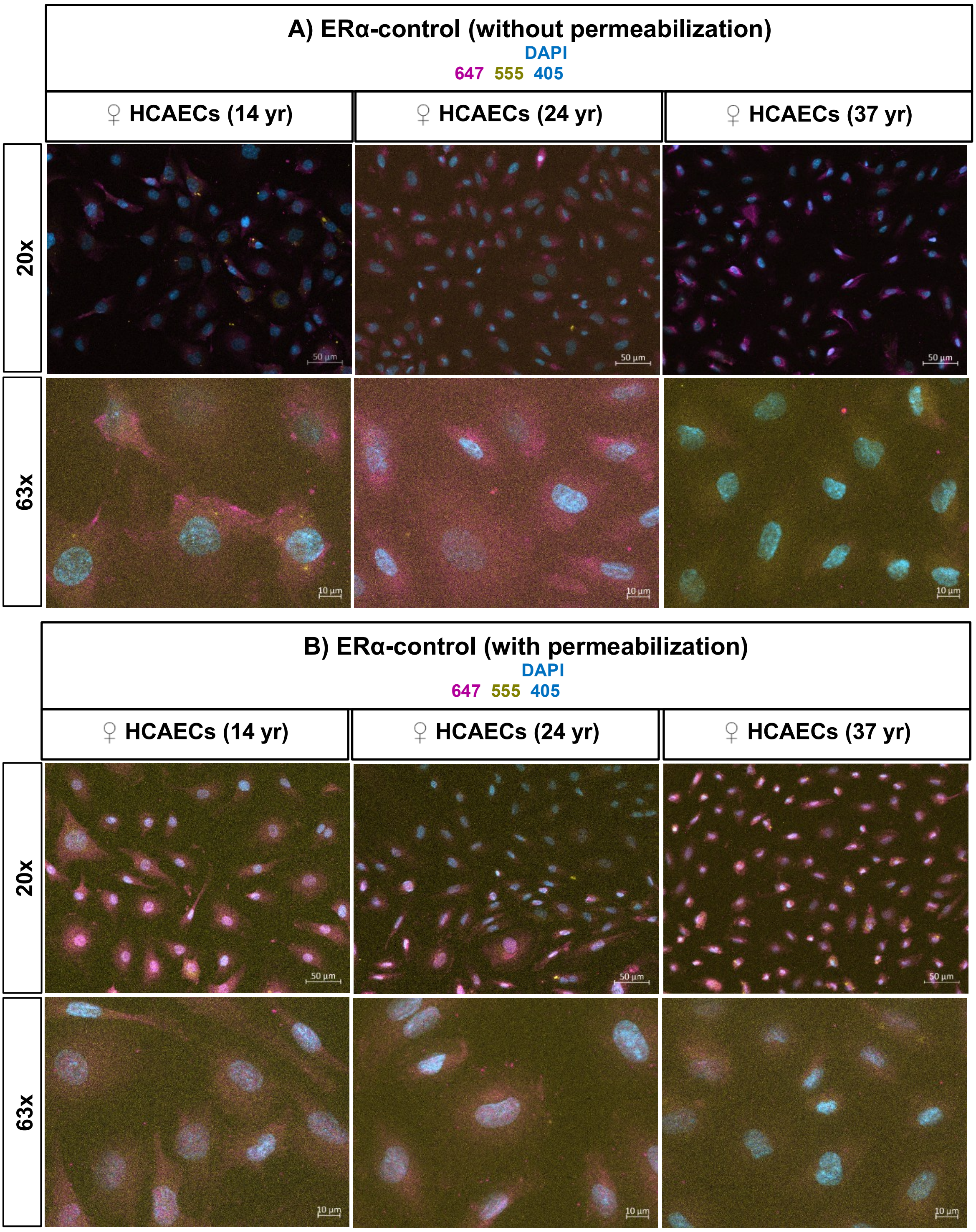

Appendix, Figure 7: Control Immunofluorescence for ERα in Female HCAECs
Merged images of HCAECs from 14, 24, and 37-year-old donors, stained with Alexa Fluor 647 (magenta), Alexa Fluor 555 (yellow), and DAPI (blue). Panel (A) shows non-permeabilized, and Panel (B) shows permeabilized samples.

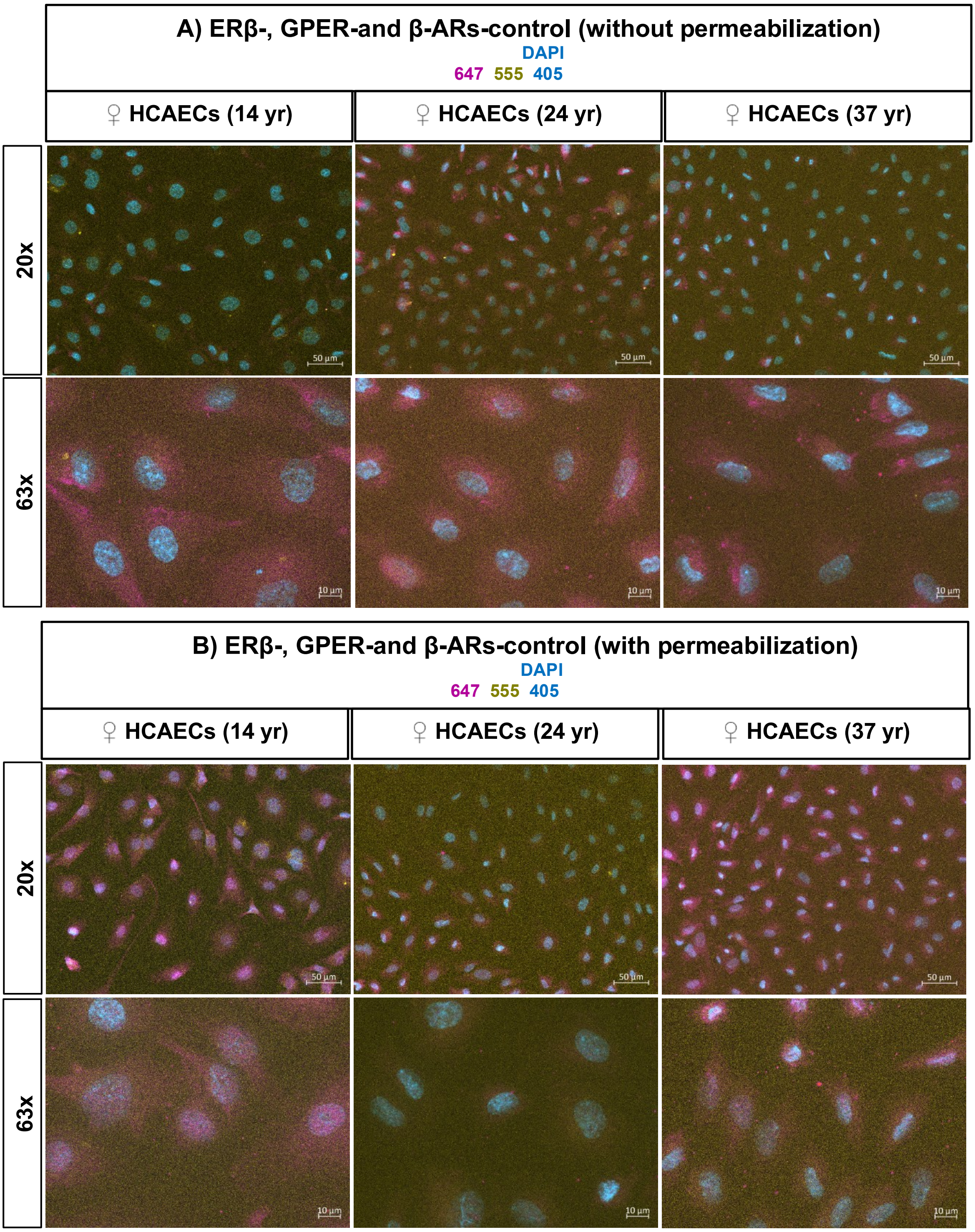

Appendix, Figure 8: Control Immunofluorescence for ERβ, GPER, and β-ARs in Female HCAECs
Merged images of HCAECs from donors aged 14, 24, and 37 years, stained with Alexa Fluor 647 (magenta), Alexa Fluor 555 (yellow), and DAPI (blue). Panel (A) shows non-permeabilized samples, and Panel (B) shows permeabilized samples.

A)

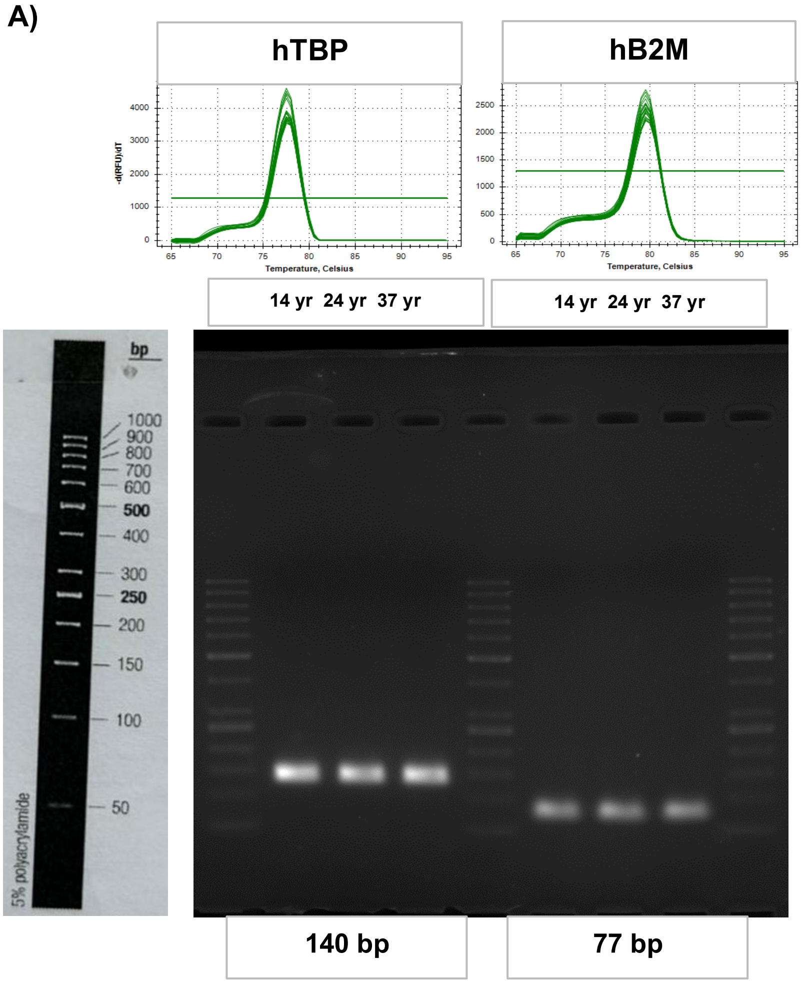
hTBP
hB2M
Temperature, Celsius
14 yr 24 yr 37 yr
14 yr 24 yr 37 yr
bp
1000
900
800
700
600
500
400
300
250
200
150
100
50
5% polyacrylamide
140 bp
77 bp

B)

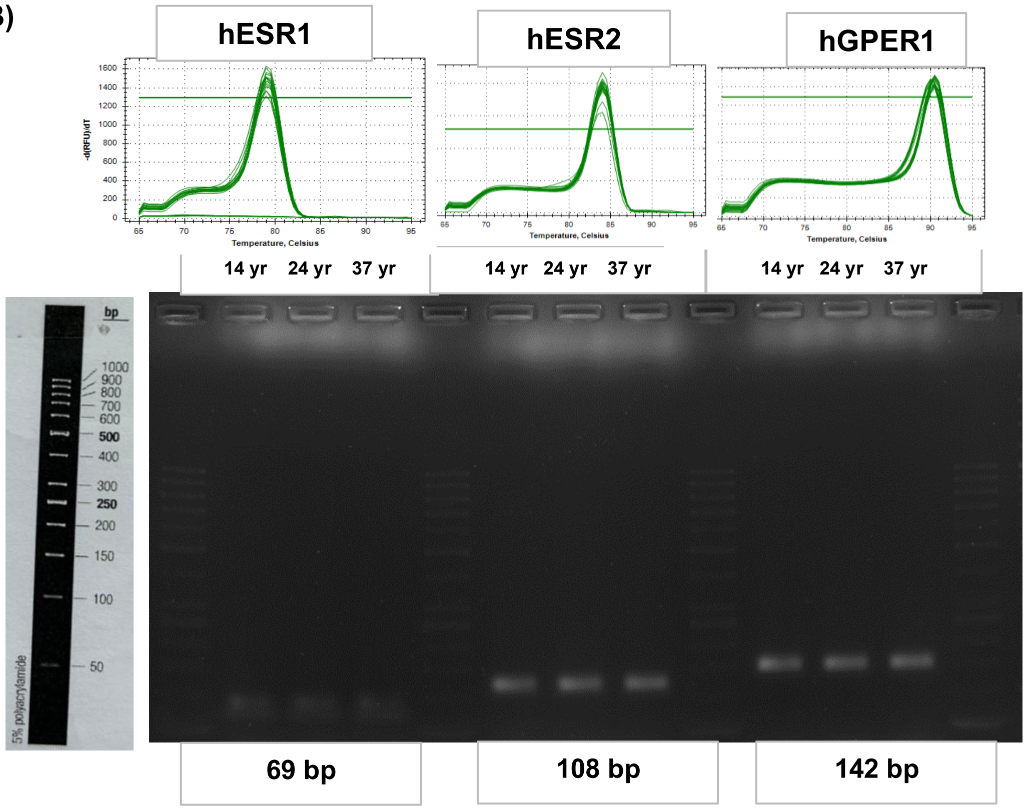
hESR1
hESR2
hGPER1
Temperature, Celsius
14 yr 24 yr 37 yr
14 yr 24 yr 37 yr
14 yr 24 yr 37 yr
bp
1000
900
800
700
600
500
400
300
250
200
150
100
50
5% polyacrylamide
69 bp
108 bp
142 bp

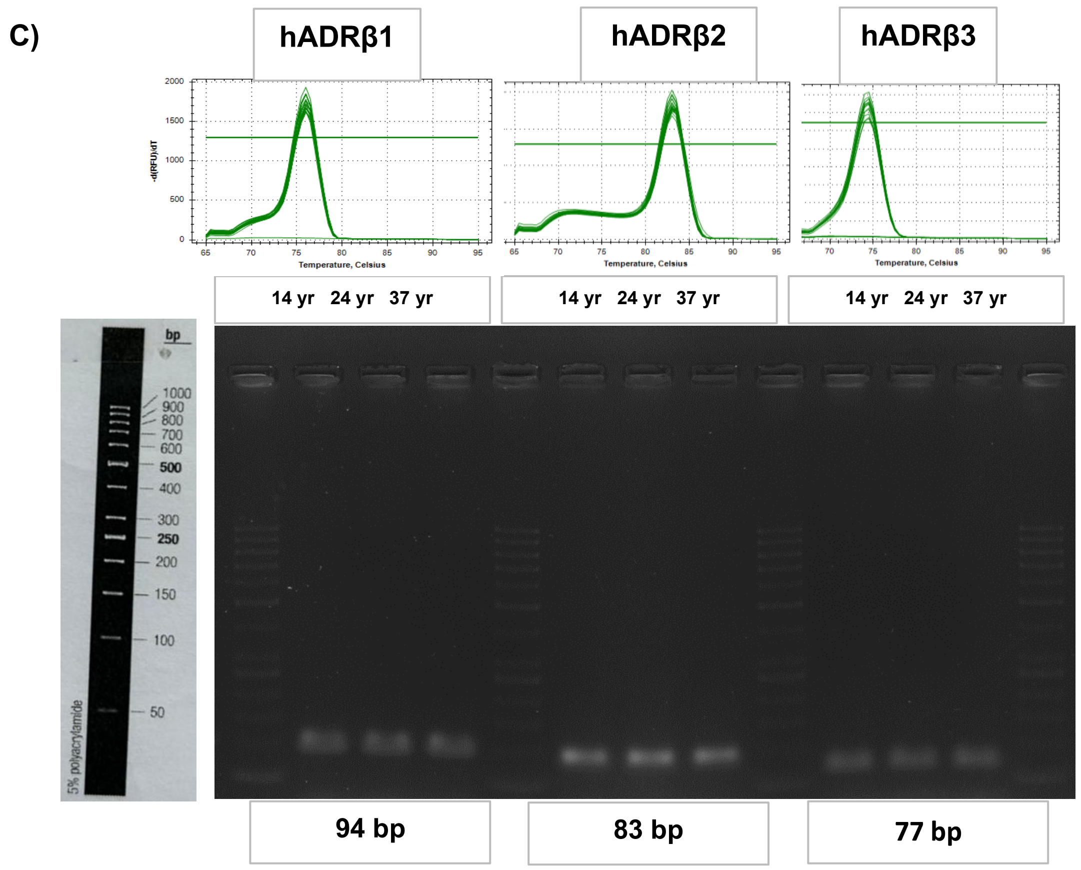

Appendix, Figure 9: Melting Curves and Agarose Gel Analysis of Reference Genes, ERs, and β-ARs in Female HCAECs

Melting curves and agarose gel electrophoresis results showing product sizes (in bp) for A) reference genes (hTBP, hB2M), B) ERs (hESR1, hESR2, hGPER1), and C) β-ARs (hADRβ1, hADRβ2, hADRβ3) in female HCAECs from donors aged 14, 24, and 37 years.
